# 3-[(1-Benzyl-1*H*-1,2,3-triazol-5-yl)methyl]-1,5-dimethyl-1,5-benzodiazepine-2,4-dione monohydrate

**DOI:** 10.1107/S1600536810005325

**Published:** 2010-02-13

**Authors:** R. Dardouri, Y. Kandri Rodi, Natalie Saffon, El Mokhtar Essassi, Seik Weng Ng

**Affiliations:** a Laboratoire de Chimie Organique Appliquée, Faculté des Sciences et Techniques, Université Sidi Mohamed Ben Abdallah, Fés, Morocco; b Service Commun Rayons-X FR2599, Université Paul Sabatier Bâtiment 2R1, 118 Route de Narbonne, Toulouse, France; c Laboratoire de Chimie Organique Hétérocyclique, Pôle de Compétences Pharmacochimie, Université Mohammed V-Agdal, BP 1014 Avenue Ibn Batout, Rabat, Morocco; d Department of Chemistry, University of Malaya, 50603 Kuala Lumpur, Malaysia

## Abstract

In the title compound, C_21_H_21_N_5_O_2_·H_2_O, the seven-membered ring adopts a boat-shaped conformation with the methine C atom as the prow. In the crystal, the water mol­ecule links adjacent mol­ecules by O—H⋯O and O—H⋯N hydrogen bonds into a zigzag chain running along the *c* axis of the monoclinic cell.

## Related literature

For the crystal structure of 1,5-dimethyl-1,5-benzodiazepin-2,4-dione, see: Mondieig *et al.* (2005 ▶). For the water-free structure of 1-benzyl-4-[(1,5-dimethyl-2,4-dioxobenzo-1,5-diazepin-3-yl)meth­yl]-1,2,3-triazole, see: Dardouri *et al.* (2010 ▶).
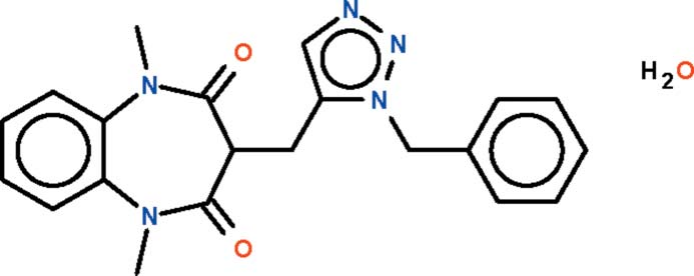


## Experimental

### 

#### Crystal data

C_21_H_21_N_5_O_2_·H_2_O
*M* *_r_* = 393.44
Monoclinic, 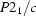

*a* = 9.6002 (1) Å
*b* = 11.9497 (2) Å
*c* = 17.0860 (2) Å
β = 92.527 (1)°
*V* = 1958.19 (4) Å^3^
*Z* = 4
Mo *K*α radiation
μ = 0.09 mm^−1^
*T* = 193 K
0.30 × 0.25 × 0.20 mm

#### Data collection

Bruker APEXII diffractometer
34134 measured reflections
4673 independent reflections
3454 reflections with *I* > 2σ(*I*)
*R*_int_ = 0.040

#### Refinement

*R*[*F* ^2^ > 2σ(*F* ^2^)] = 0.040
*wR*(*F* ^2^) = 0.108
*S* = 1.02
4673 reflections
272 parameters
2 restraints
H atoms treated by a mixture of independent and constrained refinement
Δρ_max_ = 0.24 e Å^−3^
Δρ_min_ = −0.24 e Å^−3^

### 

Data collection: *APEX2* (Bruker, 2005 ▶); cell refinement: *SAINT* (Bruker, 2005 ▶); data reduction: *SAINT*; program(s) used to solve structure: *SHELXS97* (Sheldrick, 2008 ▶); program(s) used to refine structure: *SHELXL97* (Sheldrick, 2008 ▶); molecular graphics: *X-SEED* (Barbour, 2001 ▶); software used to prepare material for publication: *publCIF* (Westrip, 2010 ▶).

## Supplementary Material

Crystal structure: contains datablocks global, I. DOI: 10.1107/S1600536810005325/bt5193sup1.cif

Structure factors: contains datablocks I. DOI: 10.1107/S1600536810005325/bt5193Isup2.hkl

Additional supplementary materials:  crystallographic information; 3D view; checkCIF report

## Figures and Tables

**Table 1:** Hydrogen-bond geometry (Å, °)

*D*—H⋯*A*	*D*—H	H⋯*A*	*D*⋯*A*	*D*—H⋯*A*
O1*W*—H1⋯O2	0.85 (1)	2.02 (1)	2.836 (2)	161 (2)
O1*W*—H2⋯N3^i^	0.85 (1)	2.10 (1)	2.937 (2)	170 (2)

Symmetry code: (i) 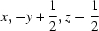.

**Fig. 1.**
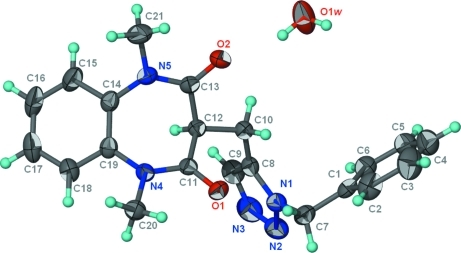
Anisotropic displacement ellipsoid plot (Barbour, 2001) of C21H21N5O2.H2O at the 70% probability level; hydrogen atoms are drawn as arbitrary radius.

## supplementary crystallographic information

### Experimental

To a solution of 1,5-dimethyl-3-propargyl-1,5-benzodiazepine-2,4-dione (8.26 × 10 ^-4^ mol) in toluene (15 ml) was added benzyl azide (9.91 × 10 ^-4^ mol). The mixture was stirred under reflux and the reaction was monitored by thin layer chromatography. On completion of the reaction, the solution was concentrated and the residue was purified by column chromatography on silica gel by using a mixture (hexane/ethyl acetate 2/1). Crystals were obtained when the solvent was allowed to evaporate.

### Refinement

Carbon-bound H atoms were placed in calculated positions (C—H 0.93–0.98 Å) and were included in the refinement in the riding model approximation, with *U*(H) set to 1.2–1.5*U*(C). The water H atoms were located in a difference Fourier map, and were refined isotropically with a distance restraint of O—H 0.84 (1) Å.

### Crystal data

**Table:**

C_21_H_21_N_5_O_2_·H_2_O	*F*(000) = 832
*M**_r_* = 393.44	*D*_x_ = 1.335 Mg m^−^^3^
Monoclinic, *P*2_1_/*c*	Mo *K*α radiation, λ = 0.71073 Å
Hall symbol: -P 2ybc	Cell parameters from 7427 reflections
*a* = 9.6002 (1) Å	θ = 2.1–28.3°
*b* = 11.9497 (2) Å	µ = 0.09 mm^−^^1^
*c* = 17.0860 (2) Å	*T* = 193 K
β = 92.527 (1)°	Block, colourless
*V* = 1958.19 (4) Å^3^	0.30 × 0.25 × 0.20 mm
*Z* = 4	

### Data collection

**Table:**

Bruker APEXII diffractometer	3454 reflections with *I* > 2σ(*I*)
Radiation source: fine-focus sealed tube	*R*_int_ = 0.040
graphite	θ_max_ = 27.5°, θ_min_ = 2.7°
φ and ω scans	*h* = −12→12
34134 measured reflections	*k* = −15→15
4673 independent reflections	*l* = −22→22

### Refinement

**Table:**

Refinement on *F*^2^	Primary atom site location: structure-invariant direct methods
Least-squares matrix: full	Secondary atom site location: difference Fourier map
*R*[*F*^2^ > 2σ(*F*^2^)] = 0.040	Hydrogen site location: inferred from neighbouring sites
*wR*(*F*^2^) = 0.108	H atoms treated by a mixture of independent and constrained refinement
*S* = 1.02	*w* = 1/[σ^2^(*F*_o_^2^) + (0.0487*P*)^2^ + 0.4622*P*] where *P* = (*F*_o_^2^ + 2*F*_c_^2^)/3
4673 reflections	(Δ/σ)_max_ = 0.001
272 parameters	Δρ_max_ = 0.24 e Å^−^^3^
2 restraints	Δρ_min_ = −0.24 e Å^−^^3^

### Fractional atomic coordinates and isotropic or equivalent isotropic displacement parameters (Å^2^)

**Table:**

	*x*	*y*	*z*	*U*_iso_*/*U*_eq_	
O1	0.47644 (10)	0.40995 (8)	0.56589 (5)	0.0303 (2)	
O2	0.16229 (11)	0.56388 (8)	0.45303 (5)	0.0338 (2)	
O1W	0.02018 (13)	0.44707 (13)	0.32908 (8)	0.0618 (4)	
H1	0.0749 (19)	0.4689 (17)	0.3660 (9)	0.072 (7)*	
H2	0.065 (2)	0.4105 (16)	0.2961 (10)	0.075 (7)*	
N1	0.27924 (13)	0.18848 (9)	0.59919 (6)	0.0300 (3)	
N2	0.25416 (14)	0.11731 (11)	0.65838 (7)	0.0400 (3)	
N3	0.15632 (15)	0.16314 (12)	0.69916 (8)	0.0440 (3)	
N4	0.45766 (11)	0.57194 (9)	0.63391 (6)	0.0257 (2)	
N5	0.22860 (12)	0.68505 (9)	0.54874 (7)	0.0303 (3)	
C1	0.31772 (15)	0.11347 (11)	0.46609 (8)	0.0293 (3)	
C2	0.39626 (18)	0.11496 (14)	0.39956 (10)	0.0438 (4)	
H2A	0.4877	0.1456	0.4026	0.053*	
C3	0.3431 (2)	0.07249 (16)	0.32909 (10)	0.0550 (5)	
H3	0.3981	0.0740	0.2842	0.066*	
C4	0.2105 (2)	0.02797 (15)	0.32374 (10)	0.0480 (4)	
H4	0.1741	−0.0014	0.2754	0.058*	
C5	0.13109 (17)	0.02632 (13)	0.38883 (9)	0.0396 (4)	
H5	0.0396	−0.0041	0.3853	0.047*	
C6	0.18408 (16)	0.06887 (12)	0.45964 (9)	0.0341 (3)	
H6	0.1283	0.0675	0.5042	0.041*	
C7	0.38193 (15)	0.15738 (12)	0.54260 (9)	0.0343 (3)	
H7A	0.4442	0.0993	0.5661	0.041*	
H7B	0.4398	0.2236	0.5316	0.041*	
C8	0.19589 (14)	0.28012 (11)	0.60124 (8)	0.0270 (3)	
C9	0.11891 (17)	0.26208 (14)	0.66575 (9)	0.0376 (3)	
H9	0.0500	0.3114	0.6842	0.045*	
C10	0.19235 (14)	0.37276 (11)	0.54245 (7)	0.0262 (3)	
H10A	0.0954	0.3825	0.5214	0.031*	
H10B	0.2503	0.3515	0.4983	0.031*	
C11	0.40317 (13)	0.48442 (10)	0.59142 (7)	0.0237 (3)	
C12	0.24519 (13)	0.48458 (11)	0.57649 (7)	0.0238 (3)	
H12	0.1999	0.4983	0.6272	0.029*	
C13	0.20750 (13)	0.58015 (11)	0.52044 (8)	0.0260 (3)	
C14	0.26507 (14)	0.70923 (11)	0.62872 (8)	0.0285 (3)	
C15	0.19149 (16)	0.79278 (12)	0.66644 (10)	0.0406 (4)	
H15	0.1132	0.8267	0.6401	0.049*	
C16	0.23055 (17)	0.82671 (13)	0.74123 (10)	0.0458 (4)	
H16	0.1806	0.8847	0.7657	0.055*	
C17	0.34254 (18)	0.77633 (14)	0.78060 (9)	0.0429 (4)	
H17	0.3707	0.8005	0.8319	0.052*	
C18	0.41355 (16)	0.69081 (12)	0.74538 (8)	0.0342 (3)	
H18	0.4889	0.6551	0.7733	0.041*	
C19	0.37605 (14)	0.65595 (11)	0.66908 (7)	0.0262 (3)	
C20	0.60870 (14)	0.57233 (12)	0.65173 (9)	0.0326 (3)	
H20A	0.6558	0.5310	0.6110	0.049*	
H20B	0.6285	0.5366	0.7026	0.049*	
H20C	0.6427	0.6497	0.6535	0.049*	
C21	0.19423 (18)	0.77880 (13)	0.49550 (10)	0.0433 (4)	
H21A	0.2198	0.7593	0.4423	0.065*	
H21B	0.2461	0.8455	0.5131	0.065*	
H21C	0.0939	0.7940	0.4957	0.065*	

### Atomic displacement parameters (Å^2^)

**Table:**

	*U*^11^	*U*^22^	*U*^33^	*U*^12^	*U*^13^	*U*^23^
O1	0.0280 (5)	0.0271 (5)	0.0357 (5)	0.0058 (4)	0.0003 (4)	−0.0025 (4)
O2	0.0347 (6)	0.0368 (6)	0.0290 (5)	0.0067 (4)	−0.0072 (4)	−0.0011 (4)
O1W	0.0327 (7)	0.0899 (11)	0.0629 (8)	−0.0016 (7)	0.0039 (6)	−0.0415 (8)
N1	0.0340 (7)	0.0267 (6)	0.0289 (6)	0.0004 (5)	−0.0036 (5)	0.0020 (5)
N2	0.0487 (8)	0.0347 (7)	0.0357 (6)	−0.0049 (6)	−0.0073 (6)	0.0083 (6)
N3	0.0497 (9)	0.0464 (8)	0.0356 (7)	−0.0104 (7)	−0.0001 (6)	0.0081 (6)
N4	0.0225 (6)	0.0286 (6)	0.0257 (5)	0.0015 (4)	−0.0009 (4)	−0.0022 (5)
N5	0.0319 (6)	0.0249 (6)	0.0336 (6)	0.0048 (5)	−0.0035 (5)	0.0013 (5)
C1	0.0335 (8)	0.0185 (6)	0.0359 (7)	0.0047 (5)	0.0022 (6)	0.0012 (5)
C2	0.0440 (10)	0.0384 (9)	0.0498 (9)	−0.0047 (7)	0.0128 (7)	−0.0010 (7)
C3	0.0680 (13)	0.0574 (11)	0.0410 (9)	−0.0038 (9)	0.0197 (9)	−0.0051 (8)
C4	0.0634 (12)	0.0453 (10)	0.0348 (8)	0.0042 (8)	−0.0030 (8)	−0.0043 (7)
C5	0.0391 (9)	0.0356 (8)	0.0434 (8)	0.0008 (7)	−0.0051 (7)	−0.0024 (7)
C6	0.0359 (8)	0.0315 (7)	0.0352 (7)	0.0013 (6)	0.0034 (6)	0.0009 (6)
C7	0.0305 (8)	0.0281 (7)	0.0440 (8)	0.0059 (6)	−0.0021 (6)	−0.0030 (6)
C8	0.0267 (7)	0.0259 (7)	0.0281 (6)	−0.0022 (5)	−0.0019 (5)	−0.0035 (5)
C9	0.0380 (8)	0.0407 (8)	0.0343 (7)	−0.0040 (7)	0.0036 (6)	0.0006 (7)
C10	0.0256 (7)	0.0259 (6)	0.0267 (6)	0.0005 (5)	−0.0019 (5)	−0.0018 (5)
C11	0.0265 (7)	0.0238 (6)	0.0206 (6)	0.0020 (5)	−0.0001 (5)	0.0026 (5)
C12	0.0230 (6)	0.0252 (6)	0.0233 (6)	0.0024 (5)	0.0005 (5)	−0.0012 (5)
C13	0.0209 (6)	0.0279 (7)	0.0291 (6)	0.0043 (5)	0.0001 (5)	−0.0008 (5)
C14	0.0276 (7)	0.0232 (6)	0.0350 (7)	−0.0022 (5)	0.0027 (5)	−0.0035 (5)
C15	0.0317 (8)	0.0308 (8)	0.0592 (10)	0.0033 (6)	0.0016 (7)	−0.0127 (7)
C16	0.0397 (9)	0.0375 (9)	0.0612 (10)	−0.0042 (7)	0.0145 (8)	−0.0241 (8)
C17	0.0465 (10)	0.0447 (9)	0.0382 (8)	−0.0120 (8)	0.0092 (7)	−0.0174 (7)
C18	0.0367 (8)	0.0355 (8)	0.0303 (7)	−0.0053 (6)	0.0009 (6)	−0.0045 (6)
C19	0.0262 (7)	0.0250 (6)	0.0278 (6)	−0.0028 (5)	0.0049 (5)	−0.0032 (5)
C20	0.0246 (7)	0.0345 (8)	0.0384 (7)	0.0003 (6)	−0.0031 (6)	−0.0033 (6)
C21	0.0497 (10)	0.0293 (8)	0.0497 (9)	0.0043 (7)	−0.0098 (7)	0.0087 (7)

### Geometric parameters (Å, °)

**Table:**

O1—C11	1.2263 (15)	C7—H7A	0.9900
O2—C13	1.2283 (15)	C7—H7B	0.9900
O1W—H1	0.85 (1)	C8—C9	1.371 (2)
O1W—H2	0.85 (1)	C8—C10	1.4942 (18)
N1—N2	1.3511 (16)	C9—H9	0.9500
N1—C8	1.3576 (17)	C10—C12	1.5346 (18)
N1—C7	1.4591 (19)	C10—H10A	0.9900
N2—N3	1.314 (2)	C10—H10B	0.9900
N3—C9	1.354 (2)	C11—C12	1.5269 (18)
N4—C11	1.3642 (16)	C12—C13	1.5237 (18)
N4—C19	1.4227 (17)	C12—H12	1.0000
N4—C20	1.4685 (17)	C14—C15	1.3968 (19)
N5—C13	1.3556 (17)	C14—C19	1.3970 (19)
N5—C14	1.4252 (17)	C15—C16	1.377 (2)
N5—C21	1.4714 (18)	C15—H15	0.9500
C1—C6	1.389 (2)	C16—C17	1.381 (3)
C1—C2	1.392 (2)	C16—H16	0.9500
C1—C7	1.5142 (19)	C17—C18	1.381 (2)
C2—C3	1.383 (2)	C17—H17	0.9500
C2—H2A	0.9500	C18—C19	1.4006 (18)
C3—C4	1.379 (3)	C18—H18	0.9500
C3—H3	0.9500	C20—H20A	0.9800
C4—C5	1.376 (2)	C20—H20B	0.9800
C4—H4	0.9500	C20—H20C	0.9800
C5—C6	1.388 (2)	C21—H21A	0.9800
C5—H5	0.9500	C21—H21B	0.9800
C6—H6	0.9500	C21—H21C	0.9800
			
H1—O1W—H2	110 (2)	C8—C10—H10B	109.0
N2—N1—C8	111.33 (12)	C12—C10—H10B	109.0
N2—N1—C7	118.92 (12)	H10A—C10—H10B	107.8
C8—N1—C7	129.70 (12)	O1—C11—N4	122.22 (12)
N3—N2—N1	106.85 (12)	O1—C11—C12	121.50 (11)
N2—N3—C9	108.83 (12)	N4—C11—C12	116.28 (11)
C11—N4—C19	124.08 (11)	C13—C12—C11	108.21 (10)
C11—N4—C20	117.69 (11)	C13—C12—C10	110.43 (10)
C19—N4—C20	117.86 (11)	C11—C12—C10	111.81 (10)
C13—N5—C14	123.78 (11)	C13—C12—H12	108.8
C13—N5—C21	117.24 (11)	C11—C12—H12	108.8
C14—N5—C21	118.51 (11)	C10—C12—H12	108.8
C6—C1—C2	118.15 (14)	O2—C13—N5	121.46 (12)
C6—C1—C7	122.74 (13)	O2—C13—C12	122.34 (12)
C2—C1—C7	119.09 (13)	N5—C13—C12	116.19 (11)
C3—C2—C1	120.93 (16)	C15—C14—C19	119.22 (13)
C3—C2—H2A	119.5	C15—C14—N5	118.65 (13)
C1—C2—H2A	119.5	C19—C14—N5	122.05 (12)
C4—C3—C2	120.21 (16)	C16—C15—C14	121.08 (15)
C4—C3—H3	119.9	C16—C15—H15	119.5
C2—C3—H3	119.9	C14—C15—H15	119.5
C5—C4—C3	119.64 (16)	C15—C16—C17	119.87 (14)
C5—C4—H4	120.2	C15—C16—H16	120.1
C3—C4—H4	120.2	C17—C16—H16	120.1
C4—C5—C6	120.30 (16)	C16—C17—C18	119.93 (14)
C4—C5—H5	119.8	C16—C17—H17	120.0
C6—C5—H5	119.8	C18—C17—H17	120.0
C5—C6—C1	120.76 (14)	C17—C18—C19	120.94 (14)
C5—C6—H6	119.6	C17—C18—H18	119.5
C1—C6—H6	119.6	C19—C18—H18	119.5
N1—C7—C1	113.52 (12)	C14—C19—C18	118.89 (13)
N1—C7—H7A	108.9	C14—C19—N4	122.37 (11)
C1—C7—H7A	108.9	C18—C19—N4	118.64 (12)
N1—C7—H7B	108.9	N4—C20—H20A	109.5
C1—C7—H7B	108.9	N4—C20—H20B	109.5
H7A—C7—H7B	107.7	H20A—C20—H20B	109.5
N1—C8—C9	103.48 (12)	N4—C20—H20C	109.5
N1—C8—C10	125.18 (12)	H20A—C20—H20C	109.5
C9—C8—C10	131.30 (13)	H20B—C20—H20C	109.5
N3—C9—C8	109.50 (14)	N5—C21—H21A	109.5
N3—C9—H9	125.2	N5—C21—H21B	109.5
C8—C9—H9	125.2	H21A—C21—H21B	109.5
C8—C10—C12	113.14 (10)	N5—C21—H21C	109.5
C8—C10—H10A	109.0	H21A—C21—H21C	109.5
C12—C10—H10A	109.0	H21B—C21—H21C	109.5
			
C8—N1—N2—N3	−0.75 (15)	N4—C11—C12—C10	−169.06 (10)
C7—N1—N2—N3	−178.41 (12)	C8—C10—C12—C13	−166.63 (11)
N1—N2—N3—C9	0.48 (16)	C8—C10—C12—C11	72.82 (14)
C6—C1—C2—C3	−0.4 (2)	C14—N5—C13—O2	172.36 (12)
C7—C1—C2—C3	178.00 (15)	C21—N5—C13—O2	0.4 (2)
C1—C2—C3—C4	0.1 (3)	C14—N5—C13—C12	−8.58 (19)
C2—C3—C4—C5	0.2 (3)	C21—N5—C13—C12	179.44 (12)
C3—C4—C5—C6	−0.2 (3)	C11—C12—C13—O2	111.69 (14)
C4—C5—C6—C1	−0.1 (2)	C10—C12—C13—O2	−10.99 (18)
C2—C1—C6—C5	0.5 (2)	C11—C12—C13—N5	−67.36 (14)
C7—C1—C6—C5	−177.93 (13)	C10—C12—C13—N5	169.96 (11)
N2—N1—C7—C1	101.20 (14)	C13—N5—C14—C15	−132.02 (15)
C8—N1—C7—C1	−75.96 (18)	C21—N5—C14—C15	39.86 (19)
C6—C1—C7—N1	−22.18 (19)	C13—N5—C14—C19	51.2 (2)
C2—C1—C7—N1	159.45 (13)	C21—N5—C14—C19	−136.95 (14)
N2—N1—C8—C9	0.69 (15)	C19—C14—C15—C16	2.9 (2)
C7—N1—C8—C9	178.03 (13)	N5—C14—C15—C16	−173.96 (14)
N2—N1—C8—C10	−177.24 (12)	C14—C15—C16—C17	−1.2 (3)
C7—N1—C8—C10	0.1 (2)	C15—C16—C17—C18	−1.1 (2)
N2—N3—C9—C8	−0.06 (17)	C16—C17—C18—C19	1.7 (2)
N1—C8—C9—N3	−0.38 (16)	C15—C14—C19—C18	−2.3 (2)
C10—C8—C9—N3	177.37 (13)	N5—C14—C19—C18	174.49 (12)
N1—C8—C10—C12	−113.56 (14)	C15—C14—C19—N4	−178.56 (13)
C9—C8—C10—C12	69.12 (19)	N5—C14—C19—N4	−1.8 (2)
C19—N4—C11—O1	−175.58 (12)	C17—C18—C19—C14	0.0 (2)
C20—N4—C11—O1	−2.71 (18)	C17—C18—C19—N4	176.42 (13)
C19—N4—C11—C12	5.01 (17)	C11—N4—C19—C14	−46.74 (18)
C20—N4—C11—C12	177.88 (11)	C20—N4—C19—C14	140.41 (13)
O1—C11—C12—C13	−110.30 (13)	C11—N4—C19—C18	137.00 (13)
N4—C11—C12—C13	69.11 (14)	C20—N4—C19—C18	−35.85 (17)
O1—C11—C12—C10	11.53 (16)		

### Hydrogen-bond geometry (Å, °)

**Table:**

*D*—H···*A*	*D*—H	H···*A*	*D*···*A*	*D*—H···*A*
O1W—H1···O2	0.85 (1)	2.02 (1)	2.836 (2)	161 (2)
O1W—H2···N3^i^	0.85 (1)	2.10 (1)	2.937 (2)	170 (2)

Symmetry codes: (i) *x*, −*y*+1/2, *z*−1/2.
